# Reducing the risks of nuclear war: the role of health professionals^†^

**DOI:** 10.1093/jtm/taad105

**Published:** 2023-09-07

**Authors:** Andy Haines, Ira Helfand, Arun Mitra, Tilman Ruff, Chris Zielinski

**Affiliations:** London School of Hygiene and Tropical Medicine, UK; International Physicians for the Prevention of Nuclear War; International Physicians for the Prevention of Nuclear War; International Physicians for the Prevention of Nuclear War; University of Winchester, UK; World Association of Medical Editors; British Medical Journal; International Nursing Review; Medical Journal of Australia; JAMA; Dutch Journal of Medicine; Chinese Science Bulletin; The Lancet; African Journal of Primary Health Care & Family Medicine; Revista de Saúde Pública; Journal of Public Health Policy; New England Journal of Medicine; National Medical Journal of India; African Health Sciences; East African Medical Journal

In January 2023, the Science and Security Board of the Bulletin of the Atomic Scientists moved the hands of the Doomsday Clock forward to 90 s before midnight, reflecting the growing risk of nuclear war.^1^ In August 2022, the UN Secretary-General, António Guterres warned that the world is now in ‘a time of nuclear danger not seen since the height of the Cold War’.^2^ The danger has been underlined by growing tensions between many nuclear armed states.^1^^,^^3^ As editors of health and medical journals worldwide, we call on health professionals to alert the public and our leaders to this major danger to public health and the essential life support systems of the planet—and urge action to prevent it.

Current nuclear arms control and non-proliferation efforts are inadequate to protect the world’s population against the threat of nuclear war by design, error, or miscalculation. The Treaty on the Non-Proliferation of Nuclear Weapons (NPT) commits each of the 190 participating nations ‘to pursue negotiations in good faith on effective measures relating to cessation of the nuclear arms race at an early date and to nuclear disarmament, and on a treaty on general and complete disarmament under strict and effective international control’.^4^ Progress has been disappointingly slow and the most recent NPT review conference in 2022 ended without an agreed statement.^5^ There are many examples of near disasters that have exposed the risks of depending on nuclear deterrence for the indefinite future.^6^ Modernisation of nuclear arsenals could increase risks: for example, hypersonic missiles decrease the time available to distinguish between an attack and a false alarm, increasing the likelihood of rapid escalation.

Any use of nuclear weapons would be catastrophic for humanity. Even a ‘limited’ nuclear war involving only 250 of the 13 000 nuclear weapons in the world could kill 120 million people outright and cause global climate disruption leading to a nuclear famine, putting 2 billion people at risk.^7^^,^^8^ A large-scale nuclear war between the USA and Russia could kill 200 million people or more in the near term, and potentially cause a global ‘nuclear winter’ that could kill 5–6 billion people, threatening the survival of humanity.^7^^,^^8^ Once a nuclear weapon is detonated, escalation to all-out nuclear war could occur rapidly. The prevention of any use of nuclear weapons is therefore an urgent public health priority and fundamental steps must also be taken to address the root cause of the problem—by abolishing nuclear weapons.

The health community has had a crucial role in efforts to reduce the risk of nuclear war and must continue to do so in the future.^9^ In the 1980s, the efforts of health professionals, led by the International Physicians for the Prevention of Nuclear War (IPPNW), helped to end the Cold War arms race by educating policy makers and the public on both sides of the Iron Curtain about the medical consequences of nuclear war. This was recognised when the 1985 Nobel Peace Prize was awarded to the IPPNW^10^ (http://www.ippnw.org).

In 2007, the IPPNW launched the International Campaign to Abolish Nuclear Weapons, which grew into a global civil society campaign with hundreds of partner organisations. A pathway to nuclear abolition was created with the adoption of the Treaty on the Prohibition of Nuclear Weapons in 2017, for which the International Campaign to Abolish Nuclear Weapons was awarded the 2017 Nobel Peace Prize. International medical organisations, including the International Committee of the Red Cross, the IPPNW, the World Medical Association, the World Federation of Public Health Associations, and the International Council of Nurses, had key roles in the process leading up to the negotiations, and in the negotiations themselves, presenting the scientific evidence about the catastrophic health and environmental consequences of nuclear weapons and nuclear war. They continued this important collaboration during the First Meeting of the States Parties to the Treaty on the Prohibition of Nuclear Weapons, which currently has 92 signatories, including 68 member states.^11^

We now call on health professional associations to inform their members worldwide about the threat to human survival and to join with the IPPNW to support efforts to reduce the near-term risks of nuclear war, including three immediate steps on the part of nuclear-armed states and their allies: first, adopt a no first use policy^12^; second, take their nuclear weapons off hair-trigger alert; and, third, urge all states involved in current conflicts to pledge publicly and unequivocally that they will not use nuclear weapons in these conflicts. We further ask them to work for a definitive end to the nuclear threat by supporting the urgent commencement of negotiations among the nuclear-armed states for a verifiable, timebound agreement to eliminate their nuclear weapons in accordance with commitments in the NPT, opening the way for all nations to join the Treaty on the Prohibition of Nuclear Weapons.

The danger is great and growing. The nuclear armed states must eliminate their nuclear arsenals before they eliminate us. The health community played a decisive part during the Cold War and more recently in the development of the Treaty on the Prohibition of Nuclear Weapons. We must take up this challenge again as an urgent priority, working with renewed energy to reduce the risks of nuclear war and to eliminate nuclear weapons.

**Provenance and peer review**: Commissioned for the BMJ; not externally peer reviewed.

**Competing interests**: The authors have warranted to BMJ that they have read and understood the BMJ policy on declaration of interests and have no relevant interests to declare.
